# Lymphadenectomy and optimal excise lymph nodes count for early-stage primary fallopian tube cancer: a SEER-based study

**DOI:** 10.1186/s12905-023-02833-y

**Published:** 2023-12-21

**Authors:** Yuexi Liu, Fanfan Huang, Qiuying Gu, Jinlong Wang, Qingmiao Wang, Yuyang Wu, Lijuan Li, Yao Xiao

**Affiliations:** 1 Department of Obstetrics and Gynecology, The first Affiliated Hospital of Chongqing Medical university, Chongqing, China; 2 Department of Ophthalmology, The first Affiliated Hospital of Chongqing Medical university, Chongqing, China; 3 Chongqing Medical university, Address No. 1, Medical College Road, Chongqing, Yuzhong District 400016 China; 4 Department of Gynecologic Oncology, School of Medicine, the International Peace Maternity and Child Health Hospital, Shanghai Jiao Tong University, Shanghai, China

**Keywords:** Primary fallopian tube cancer, Lymphadenectomy, Optimal excise lymph node count, Prognosis, SEER database

## Abstract

**Backgrounds:**

There is still no consensus on the significance of Lymphadenectomy (LD) and the number of lymph nodes that need to be excised (ELNs) for adequate LD in patients with early-stage primary fallopian tube cancer (PFTC). Our endeavor is geared towards deepening comprehension of LD in early-stage PFTC and identify the optimal cut-off of ELNs.

**Methods:**

This SEER-based study analyzed the clinical data of patients with early-stage PFTC between 2000 and 2018. X-tile was employed to confirm the optimal cut-off of ELNs. The survival data between groups were analyzed by the Kaplan-Meier estimates, Log-rank test and Cox proportional hazards model.

**Results:**

There was significant improvement in both mean cancer-specific survival (CSS, *p* < 0.001) and overall survival (OS, *p* < 0.001) in LD group. Regardless of matched or not, LD was identified as an independent protective factor of CSS and OS. The optimal 3-year CSS-based cutoff of ELNs was 11 (*p* = 0.026) as determined by X-tile. Both the mean CSS (*p* = 0.001) and mean OS (*p* = 0.002) in adequate LD group (ELNs > 11, *n* = 574) were significantly longer than these in inadequate LD group (ELNs ≤ 11, *n* = 738). Adequate LD, FIGO stage, tumor grade and histology were significant prognostic factors for CSS and OS.

**Conclusion:**

LD is an independent protective prognostic factor of patients with early-stage PFTC. The association between ELNs > 11 and an improved prognosis is evident. Future studies are needed to further clarify the results above.

**Supplementary Information:**

The online version contains supplementary material available at 10.1186/s12905-023-02833-y.

## Introduction

Primary fallopian tube cancer (PFTC) is an uncommon yet formidable malignancy, accounting for a minority (0.14–1.8%) of female genital malignancies [1]. Recent evidence strongly suggests that a substantial proportion of tumors traditionally classified as high-grade serous carcinoma of the ovary or peritoneum may have their origins in the fallopian tube [2]. Consequently, it is plausible that the true incidence of PFTC is significantly underestimated, warranting heightened awareness and attention to this disease.

Presently, PFTC is frequently conjoined with EOC due to shared clinicopathologic characteristics. However, several distinct differences should be emphasized since they develop in two separate organs with different anatomic structure and embryologic origin [3]. PFTC commonly manifests at an earlier stage (FIGO stage I/II) compared to its ovarian counterparts [4]. Furthermore, this neoplasm exhibits a heightened propensity for nodal metastasis, which significantly correlates with an unfavorable prognosis in advanced stages [5]. Notably, distant lymph node metastasis might manifest as the inaugural presentation of PFTC or serve as the primary symptom in instances of recurrence [6].

The impact of lymphadenectomy (LD) for ovarian cancer (OC) has been a subject of intense research in the past two decades [7, 8]. The extensive LION trial, a large-scale prospective study, recently reported a lack of survival benefits associated with LD in patients with advanced OC, prompting a revision of existing guidelines [9]. Presently, a multi-center prospective trial is underway in China, aiming to elucidate the implications of LD in early-stage OC [10]. However, the rarity of PFTC has resulted in a paucity of research addressing the relevance of LD, particularly in patients with early-stage disease. Additionally, there is a notable absence of published data regarding the optimal number of lymph nodes to be excised (ELNs) for an adequate LD in the context of PFTC.

In consideration of these factors, our objective was to enhance comprehension of LD in early-stage PFTC and determine the optimal ELNs cut-off. To achieve this, we leveraged data from the Surveillance, Epidemiology, and End Results (SEER) program.

## Methods

### Data resource

Utilizing the SEER Research Plus Data repository, we retrieved demographic, clinicopathologic, and survival data pertaining to individuals with the primary site designation of “primary fallopian tube” as per SEER*Stat, version 8.3.9.2. All data accessed were publicly available, de-identified, and exempt from Institutional Review Board scrutiny.

### Study population

A total of 6904 patients with histologically confirmed PFTC between 2000 and 2018 were identified initially. Exclusion criteria were applied as follows: (1) absence of primary surgery (*N* = 72); (2) FIGO stage III/IV or unknown (*N* = 4,851); (3) non-epithelial tumors (*N* = 5); (4) unknown number of excised lymph nodes (*N* = 27). Staging information for each patient was ascertained from the American Joint Cancer Committee (AJCC) and determined using the staging criteria of the International Federation of Gynecologists and Obstetricians (FIGO).

### Clinicopathological factors

The following covariates were extracted from the SEER database: age, year of diagnosis, race, FIGO stage, tumor grade, laterality, histopathology, tumor size, ELNs, chemotherapy and radiotherapy. Outcome variables encompassed vital status and the time-to-event, measured from the date of diagnosis until death, censoring, or the last follow-up, as corroborated by the SEER Program’s vital status determination. The primary endpoint was cancer-specific survival (CSS), defined as the duration from the date of diagnosis to death caused by PFTC. The secondary endpoint was overall survival (OS), denoting the interval from diagnosis to death from any cause.

### Statistical analysis

In this study, differences in baseline covariates between groups were adjusted using the inverse probability of treatment weighting (IPTW) method, which mimics a situation that treatments were randomly allocated to individuals through weighing. In short, IPTW involves two main steps. First, the probability, or propensity of being exposed is calculated. This is also called the propensity score. Second, weights for each individual are calculated as the inverse of the probability of receiving her actual exposure level. The application of these weights to the study population creates a pseudo population in which measured confounders are equally distributed across groups. As the weighting creates a pseudo population containing ‘replications’ of individuals, the sample size will be artificially inflated [11]. Consequently, the number of both NLD and LD groups in the IPTW cohort is larger than in the unmatched groups.

X-tile was used to confirm the relationship between long-term outcome and ELNs based on the projection of each possible cut-off point. Following the identification of the optimal cut-off value, patients were stratified into two groups for subsequent analysis. patients who underwent LD with ELNs surpassing the optimal cut-off were classified as the adequate lymphadenectomy (ALD) group, while those with ELNs less than or equal to the optimal cut-off were assigned to the inadequate lymphadenectomy (IALD) group.

Categorical variables were presented as numbers (percentages), while continuous variables were expressed as means (SD). Correlations among patients’ baseline characteristics were assessed using Pearson χ2, Fisher’s exact test, or the Student t-test. Additionally, standardized mean differences (SMDs) were computed for group comparisons, with SMDs less than 0.2 indicative of small differences between groups [12]. Median survival data were unavailable when the incidence of the endpoint did not reach 50%; therefore, mean survival data were analyzed using Kaplan-Meier estimates and compared through the Log-rank test. Potential risk factors were scrutinized using the Cox proportional hazards model to ascertain independent prognostic factors and their covariate-adjusted effects. All computations were executed using R 4.0.6 software, adopting a two-tailed model, and a two-sided *P* value < 0.05 in all statistical hypothesis testing was deemed statistically significant.

## Results

### Population

**Fig. 1:**
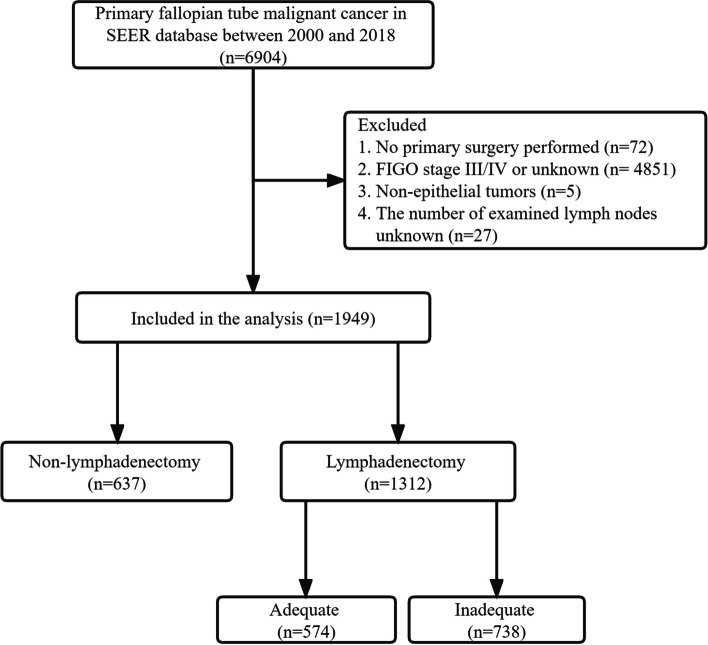
Flowchart of patients for analysis (by PowerPoint)

As illustrated in Fig. 1, a total of 1,949 patients diagnosed with FIGO stage I/II PFTC who met the criteria were ultimately included in the analysis. Among these, 1,312 patients underwent LD (LD group), while 637 did not undergo LD (NLD group). Table 1 provides a summary of the baseline characteristics, comparing groups with and without matching, utilizing the IPTW model. Examination of Table 1 reveals that majority of patients in both the NLD and LD groups exhibited high-grade tumors (63.7% in the NLD group, 71.4% in the LD group) and were diagnosed with serous carcinoma (64.1% in the NLD group, 68.8% in the LD group). Moreover, the prevalent pattern involved unilateral adnexal foci, observed in the majority of patients (91.4% in the NLD group, 95.6% in the LD group).

**Table 1:** Characteristics of patients between non-lymphadenectomy and lymphadenectomy groups in unmatched and IPTW population

		Unmatched Population N (%)		*p* value	IPTW, N (%)		*p* value	SMD
Characteristics		NLD (*N* = 637)	LD (*N* = 1312)		NLD (*N* = 1943.15)	LD (*N* = 1953.87)		
Age (mean ± SD)		64.23 ± 12.40	61.29 ± 10.84)	< 0.001	61.99 ± 12.48	62.22 ± 10.94	0.707	0.020
Race	White	531 (83.4)	1098 (83.7)	0.016	1618.6 (83.3)	1628.3 (83.3)	0.999	0.002
	Black	63 (9.9)	91 (6.9)		157.1 (8.1)	158.3 (8.1)		
	Unknown	43 (6.8)	123 (9.4)		167.4 (8.6)	167.3 (8.6)		
Grade	G1-G2	119 (18.7)	195 (14.9)	0.003	330.9 (17.0)	319.4 (16.3)	0.916	0.021
	G3-G4	406 (63.7)	937 (71.4)		1313.9 (67.6)	1339.0 (68.5)		
	Unknown	112 (17.6)	180 (13.7)		298.4 (15.4)	295.4 (15.1)		
Laterality	Unilateral	582 (91.4)	1254 (95.6)	< 0.001	1824.8 (93.9)	1832.7 (93.8)	0.926	0.004
	Bilateral	55 (8.6)	58 (4.4)		118.4 (6.1)	121.2 (6.2)		
FIGO stage	I	416 (65.3)	770 (58.7)	0.006	1198.9 (61.7)	1196.9 (61.3)	0.859	0.009
	II	221 (34.7)	542 (41.3)		744.2 (38.3)	757.0 (38.7)		
Histology	Serous	408 (64.1)	903 (68.8)	0.040	1261.9 (64.9)	1340.0 (68.6)	0.126	0.077
	Non-serous	229 (35.9)	409 (31.2)		681.2 (35.1)	613.9 (31.4)		
Tumor size	< 5 cm	400 (62.8)	733 (55.9)	< 0.001	1129.1 (58.1)	1138.2 (58.3)	0.993	0.006
	≥ 5 cm	112 (17.6)	370 (28.2)		475.7 (24.5)	479.8 (24.6)		
	Unknown	125 (19.6)	209 (15.9)		338.3 (17.4)	335.9 (17.2)		
Radiotherapy	No	626 (98.3)	1282 (97.7)	0.523	1906.9 (98.1)	1913.5 (97.9)	0.775	0.015
	Yes	11 (1.7)	30 (2.3)		36.2 (1.9)	40.4 (2.1)		
Chemotherapy	No	270 (42.4)	400 (30.5)	< 0.001	673.2 (34.6)	677.5 (34.7)	0.990	0.001
	Yes	367 (57.6)	912 (69.5)		1269.9 (65.4)	1276.4 (65.3)		

### Lymphadenectomy

To access the significance of LD, Kaplan-Meier analysis was employed to compare survival data between groups. Across all patients, the 3-year and 5- year CSS rate was 91.1% and 86.1%, the 3-year and 5- year OS rate was 88.0% and 81.3%. Within NLD group, the 3-year and 5- year CSS rate was 87.2% and 80.7%, the 3-year and 5- year OS rate was 82.4% and 72.9%. In the LD group, the corresponding rates were 93.0%, 88.7%, 90.8%, and 85.4%.

**Fig. 2:**
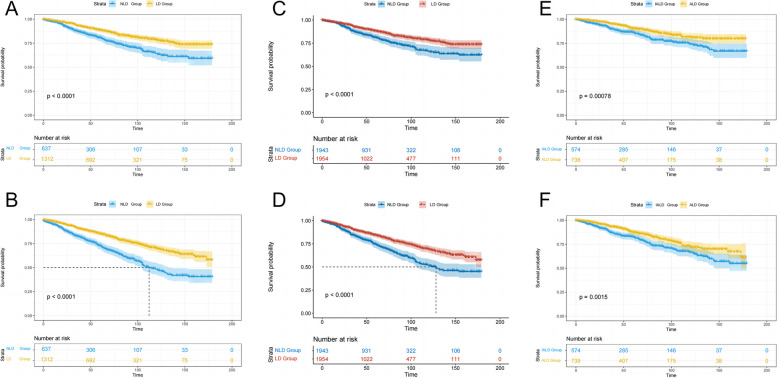
Survival between groups. **A** CSS of patients in unmatched LD group and NLD group. **B** OS of patients in unmatched LD group and NLD group. **C** CSS of patients in LD group and NLD group matched by inverse probability of treatment weighting model. **D** OS of patients in LD group and NLD group matched by inverse probability of treatment weighting model. **E** CSS of patients in ALD group and IALD group. B. OS of patients in ALD group and IALD group (by R)

Comparing the unmatched NLD group, the LD group demonstrated a substantial enhancement in both mean CSS (150.58 months vs. 132.96 months, *p* < 0.001, HR 0.561, 95% CI 0.445–0.706; Fig. 2A) and OS (140.03 months vs. 112.59 months, *p* < 0.001, HR 0.499, 95% CI 0.413–0.603; Fig. 2B). To mitigate potential baseline imbalances, we conducted further analysis in the IPTW model-matched cohort, reaffirming a significant survival benefit in the LD group (Fig. 2C and D). Moreover, irrespective of IPTW model matching, LD emerged as an independently protective factor for prognosis, as indicated by Cox proportional hazards model analyses based on CSS and OS outcomes of patients (Table S1, Table S2).

### Adequate lymphadenectomy

**Fig. 3:**
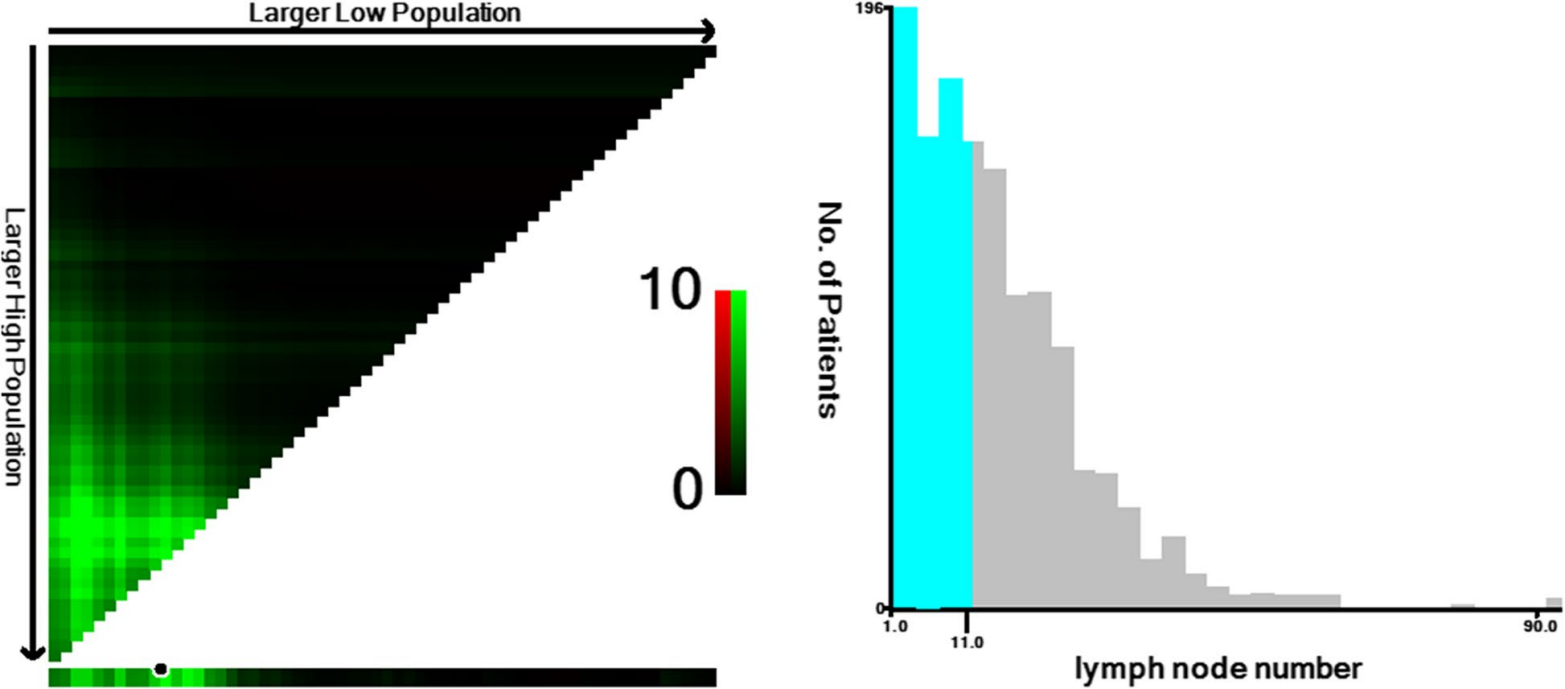
X-tile analysis of CSS. The brightest pixel represents the maximum χ2 log-rank value. The plots divided them into two groups by the cut-off point 11. The distribution of ELNs (range from 1 to 90) (by X-tile)

X-tile analysis was employed to further investigate the association between ELNs and survival within the LD group. The optimal cutoff for ELNs, based on 3-year CSS, was identified as 11 (*p* = 0.026, Fig. 3). Subsequently, patients in the LD group were categorized into two groups: ALD group, comprising patients with ELNs > 11 (*n* = 574), and IALD group, consisting of those with ELNs ≤ 11 (*n* = 738).

Table S3 presented the clinicopathological characteristics of the two groups. As observed in Table S3, patients in the ALD group were of advanced age compared to those in the IALD group, while other factors were comparable between the groups. The median number of ELNs was 6 [1–11] in the IALD group and 20 (12–90) in the ALD group.

Remarkably, both the mean CSS (155.52 months vs. 143.35 months, *p* = 0.001, HR 0.596, 95% CI 0.440–0.809; Fig. 2E) and OS (145.09 months vs. 132.85 months, *p* = 0.002, HR 0.665, 95% CI 0.513–0.856; Fig. 2F) in the ALD group were significantly prolonged compared to the IALD group. Furthermore, within the ALD group, the 3-year CSS rate and the 3-year OS rate was 95.2% and 93.3%, respectively. whereas their counterparts in the IALD group were 90.1% and 87.4%, respectively. Furthermore, subsequent analysis using the Cox proportional hazards model identified adequate LD as an independent risk factor influencing patient prognosis (Table 2).

**Table 2:** Univariate and multivariate analyses of factors affecting cancer specific survival and overall survival

		**Cancer specific survival**				**Overall survival**			
**Characteristics**	**N (%)**	**Univariate analyses**		**Multivariate analyses**		**Univariate analyses**		**Multivariate analyses**	
		***p* value**	**HR (95% CI)**	***p* value**	**HR (95% CI)**	***p* value**	**HR (95% CI)**	***p* value**	**HR (95% CI)**
**Age**		0.566				0.865			
<62	819 (62.4)		1				1		
≥62	493 (37.6)		1.095 (0.803-1.492)				0.977 (0.750-1.273)		
**Race**									
White	1098 (83.7)		1				1		
Black	91 (6.9)	0.602	1.166 (0.649-2.108)			0.154	1.397 (0.882-2.213)		
Unknown	123 (9.4)	0.633	0.861 (0.466-1.591)			0.426	0.802 (0.466-1.380)		
**Lymphadenectomy**		**0.001**		**0.001**		**0.002**		**0.002**	
Inadequate	738 (56.2)		1		1		1		1
Adequate	574 (43.8)		0.596 (0.440-0.809)		0.604 (0.445-0.819)		0.665 (0.513-0.856)		0.669 (0.518-0.864)
**Grade**									
G1-G2	195 (14.9)		1		1		1		1
G3-G4	937 (71.4)	**0.001**	2.727 (1.540-4.829)	**0.009**	2.189 (1.215-3.941)	**0.002**	1.897 (1.255-2.867)	**0.037**	1.580 (1.029-2.427)
Unknown	180 (13.7)	**0.003**	2.759 (1.429-5.328)	**0.007**	2.473 (1.275-4.795)	**0.016**	1.859 (1.125-3.070)	**0.041**	1.695 (1.022-2.811)
**Laterality**		0.752				0.719			
Unilateral	1254 (95.6)		1				1		
Bilateral	58 (4.4)		0.886 (0.356-2.110)				1.130 (0.580-2.201)		
**FIGO stage**		**0.004**		**0.031**		**0.026**		0.128	
I	770 (58.7)		1		1		1		1
II	542 (41.3)		1.568 (1.157-2.125)		1.405 (1.032-1.912)		1.340 (1.035-1.735)		1.226 (0.943-1.593)
**Histology**		**0.006**		**0.064**		**0.003**		**0.024**	
Serous	903 (68.8)		1		1		1		1
Non-serous	409 (31.2)		0.624 (0.445-0.875)		0.720 (0.509-1.019)		0.649 (0.490-0.859)		0.716 (0.536-0.956)
**Tumor size**									
<5cm	733 (55.9)		1				1		
≥5cm	370 (28.2)	0.437	1.153 (0.805-1.650)			0.596	0.920 (0.676-1.252)		
Unknown	209 (15.9)	0.088	1.394 (0.952-2.041)			0.540	1.109 (0.799-1.536)		
**Radiotherapy**		0.511				0.372			
No	1282 (97.7)		1				1		
Yes	30 (2.3)		1.314 (0.582-2.971)				1.355 (0.695-2.641)		
**Chemotherapy**		0.510				0.987			
No	400 (30.5)		1				1		
Yes	912 (69.5)		0.896 (0.646-1.242)				0.998 (0.762-1.307)		

### Univariate and multivariate analysis

Univariate analysis (Table 2) demonstrated that lymphadenectomy (LD), FIGO stage, tumor grade, and histology significantly influenced Cancer-Specific Survival (CSS) and Overall Survival (OS). Subsequently, variables exhibiting significant differences in univariate analysis were subjected to multivariate analysis. The outcomes indicated that LD and tumor grade independently served as prognostic factors for both CSS and OS. Additionally, FIGO stage emerged as an independent risk factor for CSS.

## Discussion

In accordance with prior research findings [1, 12], our study demonstrated that, akin to EOC, high-grade serous tumors predominated as the most common subtype in PFTC. Meanwhile, FIGO stage, histology and tumor grade are still important prognostic factors. However, our results revealed that patients with early-stage PFTC exhibited a more favorable survival outcome compared to early-stage EOC [13]. This observation is likely attributed to the early manifestation of symptoms related to tubal distention and the restriction of tumor dissemination by the relatively enclosed fallopian tubes [14].

Several studies have addressed the distinctive role of LD in PFTC. Bao et al. proposed that pelvic LD stood as an independent prognostic factor for survival (*p* = 0.045) [12]. In a recent retrospective study, enhanced survival was observed in PFTC patients who underwent LD [15]. Thus far, insufficient attention has been directed towards understanding the importance of LD in early-stage PFTC patients. Our results distinctly indicate that LD is correlated with a more favorable prognosis in both unmatched and well-matched patient cohorts.

The LION study established that LD has no survival benefit for advanced OC patients with normal clinical lymph nodes [9]. Several factors might account for the discrepancy between our results and those of the LION trial. Firstly, the LION trial encompassed patients with advanced-stage EOC, PFTC, or primary peritoneal cancer, lacking subgroup data based on the primary tumor site, which is essential considering the rarity of PFTC. Secondly, our study focused on those with early-stage diseases, which is different with the LION trial. Additionally, all participants in the LION trial had normal lymph nodes both preoperatively and intraoperatively, thus the early propensity of PFTC for lymph node metastasis renders these patients ineligible for the LION trial. Therefore, we contend that the significance of LN in PFTC patients is yet to be established.

A growing body of literature recognizes the correlation between ELNs and survival outcomes in various malignancies. For instance, Solomon and colleagues conducted an analysis of the SEER database involving 4,224 patients, revealing a survival benefit associated with adequate LD (ELNs ≥ 18) in patients with esophageal adenocarcinoma [16]. In the colon cancer, the National Comprehensive Cancer Network (NCCN) guidelines advocate for a minimum of 12 ELNs [17]. Similar results have been presented in bronchopulmonary carcinomas [18] and early-stage cervical cancer [19].

Despite the aforementioned significance, there remains a lack of evidence regarding the impact of the ELNs on the prognosis of early-stage PFTC. In this study, we determined the optimal cut-off for ELNs as 11 in early-stage PFTC and identifying ELNs > 11 as an independent protective prognostic factor. We extracted the clinicopathological characteristics of over 1000 patients from the SEER database, a scale of analysis that was not feasible in prior single-center retrospective studies. All patients included in this current study were diagnosed with early-stage disease, in other words, they all underwent LD and had normal lymph nodes confirmed by postoperative pathological evidence, thus ruled out the potential negative impact of lymph node metastasis on prognosis. In this context, our findings unequivocally demonstrate that patients with ELNs > 11 exhibit a superior prognosis compared to those with ELNs < 11. A possible explanation for this might be that the removal of a greater number of normal lymph nodes provides stronger evidence that tumor cells have not yet disseminated through the lymphatic system, consequently yielding a more favorable prognosis [20].

Despite these promising results, the controversy over LD in EOC and PFTC persists. on one hand, LD plays a crucial role in staging surgery, facilitating accurate tumor staging. Additionally, it serves as a surgical modality during debulking procedures to reduce tumor burden. However, the adoption of radical surgical interventions may be associated with an elevated incidence of postoperative complications, potentially leading to prolonged hospital stays and delays in the initiation of chemotherapy [7]. Previous investigations have substantiated those postoperative complications, including fever, lymph cysts, lymphedema, and peripheral sensory neurologic events, are common following LD [9, 21]. Further research is imperative to better assess and balance the procedural benefits against the adverse effects of associated complications.

### Limitation

This study has several limitations. Firstly, despite the construction of IPTW model to balance factors between groups, the presence of unknown confounders remains a potential of bias. Additionally, patients who did not undergo LD might be a subgroup of potentially advanced patients, introducing the possibility of a poorer prognosis. Secondly, the relatively low cut-off in this study might be associated with the compromised health condition or the comorbidities of the patients, limiting the feasibility of more extensive surgical procedures. Also, socioeconomic factors might affect the efficacy of surgical treatments. As factors above were not captured in our model, the results need to be interpreted with caution. Furthermore, findings from SEER database sources may not be directly applicable to patients of diverse racial and regional backgrounds. Finally, the absence of information on surgical outcomes, the extent of LD, and the patient’s baseline status could potentially affect the validity of our results.

## Conclusion

LD is an independent protective prognostic factor of patients with early-stage PFTC. The association between ELNs > 11 and an improved prognosis is evident. Future investigations, encompassing multi-center and large-sample studies, are warranted to provide additional insights into the early-stage PFTC.

### Supplementary Information

**Additional file 1:** **Table S1.** Cox proportional hazards model analyses of factors based on cancer specific survival of patients.

**Additional file 2:** **Table S2.** Cox proportional hazards model analyses of factors based on overall survival of patients.

**Additional file 3: Table S3.** Baseline characteristics of patients in adequate lymphadenectomy (ALD) and inadequate lymphadenectomy (IALD) groups.

## Data Availability

The data analyzed in this study is available at https://seer.Cancer.gov/.

## Abbreviations

PFTC: Primary fallopian tube carcinoma
EOC: Epithelial ovarian cancer
LD: Lymphadenectomy
NLD: Non-lymphadenectomy
IPTW: Inverse Probability of Treatment Weighting
OC: Ovarian cancer
ELNs: Number of excised lymph nodes
FIGO: The International Federation of Gynecologists and Obstetricians
AJCC: The American Joint Cancer Committee
CSS: Cancer-specific survival
OS: Overall survival
ALD: Adequate lymphadenectomy
IALD: Inadequate lymphadenectomy
SMDs: Standardized mean differences
