# Supplementary material for: Lymphadenectomy and optimal excise lymph nodes count for early-stage primary fallopian tube cancer: a SEER-based study
Source: BMC Womens Health. 2023 Dec 21;23:681. doi: 10.1186/s12905-023-02833-y (PMC10740229; doi:10.1186/s12905-023-02833-y)
Supplement: Supplementary file 1 — Additional file 1: Table S1. Cox proportional hazards model analyses of factors based on cancer specific survival of patients. [file 12905_2023_2833_MOESM1_ESM.docx]

**Table S1. Cox proportional hazards model analyses of factors based on cancer specific survival of patients.**

| CSS | | | | | | | | |
| --- | --- | --- | --- | --- | --- | --- | --- | --- |
| **Characteristics** | | **Unbalanced Population** | | |  | **IPTW** | | |
|  |  | **HR** | **95% CI** | ***p*-value** |  | **HR** | **95% CI** | ***p*-value** |
| Non-lymphadenectomy | | 1 |  |  |  | 1 |  |  |
| Lymphadenectomy | | 0.615 | 0.484-0.781 | **<0.001** |  | 0.611 | 0.476-0.784 | **<0.001** |
| Age, mean (SD) | | 1.044 | 1.033-1.055 | <0.001 |  | 1.036 | 1.021-1.052 | <0.001 |
| Race | White | 1 |  |  |  | 1 |  |  |
|  | Black | 1.588 | 1.064-2.372 | 0.024 |  | 1.686 | 1.114-2.550 | 0.013 |
|  | Others | 0.960 | 0.598-1.541 | 0.866 |  | 1.299 | 0.673-2.507 | 0.434 |
| Laterality | Unilateral | 1 |  |  |  | 1 |  |  |
|  | Bilateral | 1.359 | 0.832-2.222 | 0.220 |  | 1.261 | 0.751-2.118 | 0.380 |
| FIGO stage | I | 1 |  |  |  | 1 |  |  |
|  | II | 1.472 | 1.153-1.880 | 0.002 |  | 1.617 | 1.214-2.153 | 0.001 |
| Grade | G1-G2 | 1 |  |  |  | 1 |  |  |
|  | G3-G4 | 1.919 | 1.312-2.808 | <0.001 |  | 1.411 | 0.852-2.336 | 0.181 |
|  | Unknown | 1.951 | 1.255-3.036 | 0.003 |  | 1.450 | 0.875-2.403 | 0.149 |
| Histology | serous | 1 |  |  |  | 1 |  |  |
|  | non-serous | 0.764 | 0.592-0.986 | 0.039 |  | 0.802 | 0.590-1.090 | 0.158 |
| Tumor size | < 5cm | 1 |  |  |  | 1 |  |  |
|  | ≥ 5cm | 1.132 | 0.846-1.514 | 0.403 |  | 1.239 | 0.881-1.741 | 0.217 |
|  | Unknown | 1.510 | 1.134-2.012 | 0.005 |  | 1.605 | 1.181-2.183 | 0.003 |
| Chemotherapy | No | 1 |  |  |  |  |  |  |
|  | Yes | 0.895 | 0.699-1.145 | 0.376 |  | 0.855 | 0.647-1.131 | 0.272 |
| Radiotherapy | No | 1 |  |  |  | 1 |  |  |
|  | Yes | 1.238 | 0.631-2.431 | 0.534 |  | 0.981 | 0.458-2.102 | 0.961 |
