# Supplementary material for: Lymphadenectomy and optimal excise lymph nodes count for early-stage primary fallopian tube cancer: a SEER-based study
Source: BMC Womens Health. 2023 Dec 21;23:681. doi: 10.1186/s12905-023-02833-y (PMC10740229; doi:10.1186/s12905-023-02833-y)
Supplement: Supplementary file 2 — Additional file 2: Table S2. Cox proportional hazards model analyses of factors based on overall survival of patients. [file 12905_2023_2833_MOESM2_ESM.docx]

**Table S2 Cox proportional hazards model analyses of factors based on overall survival of patients.**

| OS | | | | | | | | |
| --- | --- | --- | --- | --- | --- | --- | --- | --- |
| **Characteristics** | | **Unbalanced Population** | | |  | **IPTW** | | |
|  |  | **HR** | **95%CI** | ***p*-value** |  | **HR** | **95%CI** | ***p*-value** |
| Non-lymphadenectomy | | 1 |  |  |  | 1 |  |  |
| Lymphadenectomy | | 0.593 | 0.487-0.721 | **<0.001** |  | 0.593 | 0.484-0.726 | **<0.001** |
| Age, mean (SD) | | 1.051 | 1.042-1.061 | <0.001 |  | 1.047 | 1.034-1.059 | <0.001 |
| Race | White | 1 |  |  |  | 1 |  |  |
|  | Black | 1.784 | 1.296-2.456 | <0.001 |  | 1.882 | 1.349-2.624 | <0.001 |
|  | Others | 0.935 | 0.628-1.390 | 0.737 |  | 1.175 | 0.693-1.995 | 0.549 |
| Laterality | Unilateral | 1 |  |  |  | 1 |  |  |
|  | Bilateral | 1.521 | 1.011-2.287 | 0.044 |  | 1.515 | 0.962-2.386 | 0.073 |
| FIGO stage | I | 1 |  |  |  | 1 |  |  |
|  | II | 1.281 | 1.046-1.571 | 0.017 |  | 1.374 | 1.093-1.727 | 0.007* |
| Grade | G1-G2 | 1 |  |  |  | 1 |  |  |
|  | G3-G4 | 1.486 | 1.129-1.956 | 0.005 |  | 1.237 | 0.876-1.746 | 0.227 |
|  | Unknown | 1.357 | 0.966-1.907 | 0.078 |  | 1.143 | 0.783-1.671 | 0.488 |
| Histology | serous | 1 |  |  |  | 1 |  |  |
|  | non-serous | 0.855 | 0.697-1.050 | 0.135 |  | 0.888 | 0.706-1.116 | 0.308 |
| Tumor size | < 5cm | 1 |  |  |  | 1 |  |  |
|  | ≥ 5cm | 0.986 | 0.774-1.257 | 0.910 |  | 1.088 | 0.827-1.432 | 0.547 |
|  | Unknown | 1.198 | 0.939-1.529 | 0.145 |  | 1.229 | 0.944-1.599 | 0.125 |
| Chemotherapy | No | 1 |  |  |  |  |  |  |
|  | Yes | 0.788 | 0.645-0.962 | 0.019 |  | 0.742 | 0.595-0.93 | 0.008 |
| Radiotherapy | No | 1 |  |  |  | 1 |  |  |
|  | Yes | 1.341 | 0.779-2.307 | 0.289 |  | 1.172 | 0.666-2.064 | 0.582 |
