# Supplementary material for: Lymphadenectomy and optimal excise lymph nodes count for early-stage primary fallopian tube cancer: a SEER-based study
Source: BMC Womens Health. 2023 Dec 21;23:681. doi: 10.1186/s12905-023-02833-y (PMC10740229; doi:10.1186/s12905-023-02833-y)
Supplement: Supplementary file 3 — Additional file 3: Table S3. Baseline characteristics of patients in adequate lymphadenectomy (ALD) and inadequate lymphadenectomy (IALD) groups. [file 12905_2023_2833_MOESM3_ESM.docx]

**Table S3. Baseline characteristics of patients in adequate lymphadenectomy (ALD) and inadequate lymphadenectomy (IALD) groups**

|  | | **Population N (%)** | |  |  |
| --- | --- | --- | --- | --- | --- |
| **Characteristics** | | **ALD(N=574)** | **IALD(N=738)** | ***p*-value** | **SMD** |
| Age (mean ± SD) |  | 62.12±11.48 | 60.65 ±10.26 | 0.014 | 0.135 |
| Race | White | 471 (82.1) | 627 (85.0) | 0.306 | 0.085 |
|  | Black | 46 (8.0) | 45 (6.1) |  |  |
|  | Unknown | 57 (9.9) | 66 (8.9) |  |  |
| Grade | G1-G2 | 76 (13.2) | 119 (16.1) | 0.286 | 0.088 |
|  | G3-G4 | 414 (72.1) | 523 (70.9) |  |  |
|  | Unknown | 84 (14.6) | 96 (13.0) |  |  |
| Laterality | Unilateral | 545 (94.9) | 709 (96.1) | 0.398 | 0.054 |
|  | Bilateral | 29 (5.1) | 29 (3.9) |  |  |
| FIGO stage | I | 328 (57.1) | 442 (59.9) | 0.344 | 0.056 |
|  | II | 246 (42.9) | 296 (40.1) |  |  |
| Histology | Serous | 395 (68.8) | 508 (68.8) | 1 | <0.001 |
|  | Non-serous | 179 (31.2) | 230 (31.2) |  |  |
| Tumor size | <5cm | 310 (54.0) | 423 (57.3) | 0.467 | 0.069 |
|  | ≥5cm | 167 (29.1) | 203 (27.5) |  |  |
|  | Unknown | 97 (16.9) | 112 (15.2) |  |  |
| Radiotherapy | No | 565 (98.4) | 717 (97.2) | 0.177 | 0.087 |
|  | Yes | 9 (1.6) | 21 (2.8) |  |  |
| Chemotherapy | No | 174 (30.3) | 226 (30.6) | 0.952 | 0.007 |
|  | Yes | 400 (69.7) | 512 (69.4) |  |  |
